# Assessing host range, symbiotic effectiveness, and photosynthetic rates induced by native soybean rhizobia isolated from Mozambican and South African soils

**DOI:** 10.1007/s13199-017-0520-5

**Published:** 2017-11-27

**Authors:** Cynthia Gyogluu, Mustapha Mohammed, Sanjay K. Jaiswal, Stephen Kyei-Boahen, Felix D. Dakora

**Affiliations:** 1 Department of Crop Sciences, Tshwane University of Technology, Pretoria, South Africa; 2 Department of Chemistry, Tshwane University of Technology, Private Bag X680, Pretoria, 0001 South Africa; 3 International Institute of Tropical Agriculture, Nampula, Mozambique

**Keywords:** Nodulation, N-fixed, Stomatal functioning, Whole-plant/shoot biomass, ERIC-PCR

## Abstract

Host range and cross-infectivity studies are important for identifying rhizobial strains with potential for use as inoculants. In this study, 10 native soybean rhizobia isolated from Mozambican and South African soils were evaluated for host range, symbiotic effectiveness and ability to induce high rates of photosynthesis leading to enhanced plant growth in cowpea (*Vigna unguiculata* L. Walp.), Bambara groundnut (*Vigna subterranean* L. Verdc.), Kersting’s groundnut (*Macrotyloma geocarpum* Harm) and soybean (*Glycine max* L. Merr). The test isolates had different growth rates and colony sizes. Molecular analysis based on enterobacterial repetitive intergenic consensus (ERIC)-PCR revealed high genetic diversity among the test isolates. The results further showed that isolate TUTLBC2B failed to elicit nodulation in all test plants, just as TUTNSN2A and TUTDAIAP3B were also unable to nodulate cowpea, Kersting’s bean and Bambara groundnut. Although the remaining strains formed ineffective nodules on cowpea and Kersting’s bean, they induced effective nodules on Bambara groundnut and the two soybean genotypes. Bacterial stimulation of nodule numbers, nodule dry weights and photosynthetic rates was generally greater with isolates TUTRSRH3A, TUTM19373A, TUTMCJ7B, TUTRLR3B and TUTRJN5A. As a result, these isolates elicited significantly increased accumulation of biomass in shoots and whole plants of Bambara groundnut and the two soybean genotypes. Whole-plant symbiotic nitrogen (N) of soybean and Bambara groundnut was highest for the commercial strains CB756 and WB74, as well as for TUTRLR3B, TUTMCJ7B and TUTRSRH3A, suggesting that the three native rhizobial isolates have potential for use as inoculants.

## Introduction

Some soil bacteria, collectively called rhizobia, can establish symbioses with legumes, leading to the formation of root or stem nodules. The interaction between rhizobia and legumes is of great agronomic importance and have implications for food and nutritional security as atmospheric N_2_ is reduced to NH_3_ for plant use (Herridge et al. 2008; Peoples and Craswell 1992). The legume / rhizobia symbiosis has the potential to contribute about 80% nitrogen (N) to agricultural systems (Vance 1998). This reduces the need for chemical inputs such as fertilizers, which are environmentally unsafe. The introduction of rhizobia as inoculants can enhance N nutrition in cropping systems. However, because of the poor competition of exotic rhizobia for nodule occupancy with indigenous rhizobia (Brockwell and Bottomley 1995; Vlassak et al. 1997), inoculants can fail in some environments. Rhizobia can vary widely in their ability to nodulate various legume species, with some strains showing nodulation specificity and therefore nodulating only a limited number of hosts, whiles others are highly promiscuous and can nodulate a wide range of host plants (Pueppke and Broughton 1999). Addressing the increasing decline in soil fertility for sustainable food and nutritional security in Africa requires the use of effective rhizobia capable of nodulating a wide range of host plants (Lindström et al. 2010). Furthermore, the identification of indigenous rhizobia with a wider host range that are also tolerant to abiotic stresses is important for inoculant development, especially for soils that lack effective indigenous rhizobia (Graham et al. 1994). Such active rhizobia have an added advantage of being more adapted to the soils than introduced inoculant strains. The use of microbial fertilizers is no doubt highly beneficial to smallholder farmers in Africa, given that most soils are poor in N (Adesemoye and Kloepper 2009).

In this study, indigenous rhizobia capable of forming root nodules with TGx (Tropical Glycine crosses) and non-TGx soybean plants sampled from Mozambican and South African soils were isolated and characterized phenotypically and morphologically. The isolates were also subjected to genomic fingerprinting using ERIC-PCR to establish their diversity. Ten genetically diverse isolates which also showed high N_2_-fixing efficacy on soybean were evaluated to determine their host range and cross-infectivity with four grain legume species (namely, cowpea, Bambara groundnut, Kersting’s bean, and a promiscuous (TGx) and non-promiscuous (non-TGx) soybean).

## Materials and methods

### Source of rhizobia

The strains used this study were isolated from soybean nodules collected from Nampula in Mozambique and proven to be nodule forming and N_2_-fixing bacteria using Koch’s postulates. The ten rhizobia used in this study included nine isolates from Mozambique (TUTRSRH3A, TUTM19373A, TUTNSN2A, TUTLBC2B, TUTDAIAP3B, TUTRJN5A, TUTMJM5, TUTMCJ7B, and TUTRLR3B) and one (TUTN17405) from South Africa.

### Phenotypic and molecular characterization of isolates

The isolates were cultured on YMA incubated at 28 °C to assess colony morphology. The number of days to colony appearance, colony diameter, shape and appearance/colour were also recorded for each isolate (Table 1). To assess genetic diversity among isolates, total rhizobial genomic DNA was extracted using GenElute bacterial DNA isolation kit following the manufacturer’s instructions (Sigma Aldrich, USA). The extracted genomic DNA was used to amplify intergenic repeat sequences by using enterobacterial repetitive intergenic consensus (ERIC) primers E1 5’ATGTAAGCTCCTGGGGATTCAC 3′ and E2 5’AAGTAAGTGACTGGGGTGAGCG-3′ in a 25 μl reaction volume containing 1 μl (50–80 ng) of genomic DNA, 2X PCR master mix (12.5 μl) (Bioline, USA), 1.25 μl (10 pM) of each primer and 9 μl double distilled water incubated in Thermal cycler (T100 BIO-RAD, USA) by the procedure described by de Bruijn (1992). The amplified products were separated on 1.2% agarose gel stained with ethidium bromide in Gel electrophoresis system at 85 V for 3.5 h and visualised on Gel Doc™ XR+ (BIO-RAD, USA). The banding pattern data were recorded in binary (1, 0) form and analysed using NTSYS pc 2.1 software (Rohlf 2009).

**Table 1:** Morphological description of isolates used in this study

Isolate	DTA	Colony diameter (mm)	Colony shape	Colony appearance
TUTLBC2B	4	2	round	Shiny/transparent
TUTRSRH3A	9	<1	round	Opaque
TUTM19373A	8	2	round	Creamy
TUTNSN2A	5	2	oval	Creamy
TUTN17405	4	7	round	Creamy
TUTDAIAP3B	5	1	round	Creamy
TUTRJN5A	6	2	round	Creamy
TUTMJM5	6	2	round	Shiny/transparent
TUTMCJ7B	4	3	round	Shiny/transparent
TUTRLR3B	6	<1	round	Opaque

DTA = days to colony appearance

### Seed germination for host range test

To assess the host range and symbiotic performance of the rhizobia isolates, experiments were carried out in the glasshouse using four grain legumes (namely cowpea, Kersting’s bean, Bambara groundnut, and soybean (non-promiscuous soybean PAN1664R and promiscuous soybean TGx1835-10E). The seeds of test species were rinsed in 95% ethanol for 10 s and submerged in 0.1% sodium hypochlorite (commercial bleach) for three minutes. The seeds were then rinsed in six changes of sterilised distilled water. The sterilised seeds were transferred onto petri dishes lined with sterile Whatman No 2 filter paper, and incubated to germinate at 28 °C.

### Plant culture in sterile sand

Sterile sand (Green’s Sand, Pretoria) was used as a rooting medium for the plants. After germination, one seedling was transplanted per pot. For each legume species, there were three replicate pots per isolate. A commercial inoculant of *Bradyrhizobium japonicum* strain WB74 (Stimuplant, Pretoria) was included with the soybean isolates as control, while *Bradyrhizobium* strain CB765 was used as control for the other legumes. The seedlings were supplied with Dilworth’s nutrient (Broughton and Dilworth 1971) at 3 days intervals, and watered when necessary. The daily temperatures in the glasshouse were between 25 °C and 30 °C.

### Gas-exchange measurements

For each legume species, gas-exchange measurements were done on three fully expanded trifoliate leaves per each replicate pot. Thus, a total of nine readings (from three plants) were taken per treatment for each legume species. The means of three readings obtained from each pot were used for analysis. Photosynthetic rates (A), stomatal conductance (gs), intercellular CO_2_ concentration (Ci), and transpiration rate (E) were measured on a single leaf of the nine selected plants using a portable infra-red gas analyser (LI 6400 XT, version 6.2). The prevailing conditions in the chamber included: photosynthetic photon flux density of 1000 μmolm^−2^ s^−1^, CO_2_ concentration of 380 μmolmol^−1^, gas flow of 500 μmols^−1^, and a temperature of 25 °C. Measurements were taken at 15, 25 and 35 days after planting, usually in the mornings between 8 and 10 am each day. An instantaneous measure of water-use efficiency was computed as the ratio of A to gs (Singh and Reddy 2011), and the data presented for photosynthesis, stomatal conductance and transpiration rates.

### Plant harvest for assessing nodulation and plant growth

At 45 days after planting (DAP), the seedlings were harvested and separated into shoots, roots and nodules. Nodule number per plant, nodule pigmentation, and nodule distribution on the roots were also recorded. The shoots and roots were oven-dried (60 °C) for 72 h, and weighed. The shoots were ground, (0.85 mm) for analysis of N concentration using the Kjeldahl digestion at the institute of plant production, Elsenberg. Since plants were supplied with N-free nutrient solution during growth period, shoot N accumulation (the product of %N and plant biomass) was used as a measure of N-fixed. All data collected including nodule number, nodule dry weight, shoot dry matter, and root dry weight were subjected to a 1-way ANOVA using STATISTICA 8.0 program (StatSoft 2007). Where there were significant treatment differences, the Duncan multiple range test was used to separate the means at *p* ≤ 0.05.

## Results

### Phenotypic and genetic characterization of isolates

**Fig. 1:**
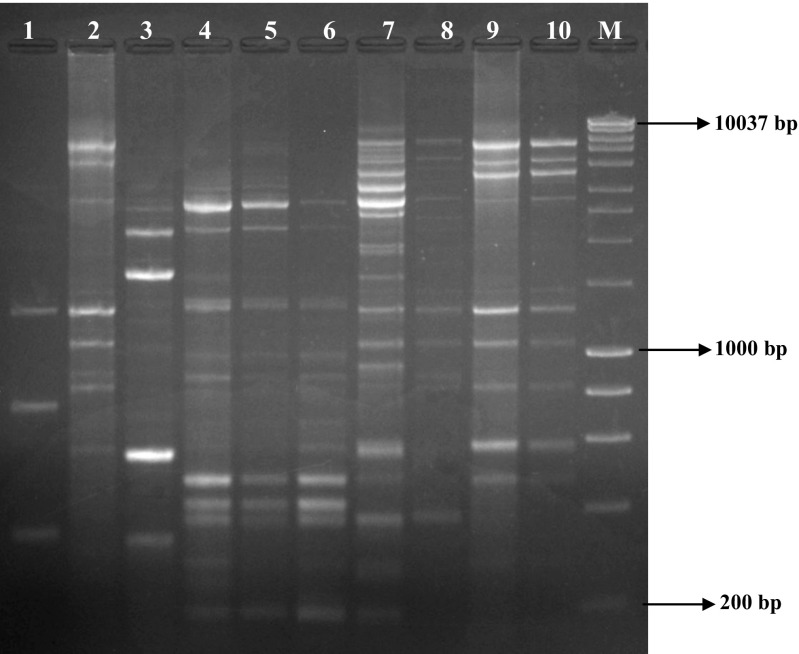
Gel image of ERIC-PCR banding pattern of soybean rhizobial isolates. 1 = TUTLBC2B; 2 = TUTRSRH3A; 3 = TUTM19373A; 4 = TUTNSN2A; 5 = TUT17405; 6 = TUTDAIAP3B; 7 = TUTRJN5A; 8 = TUTMJM5; 9 = TUTMCJ7B; 10 = TUTRLR3B; M = 1 kb ladder

**Fig. 2:**
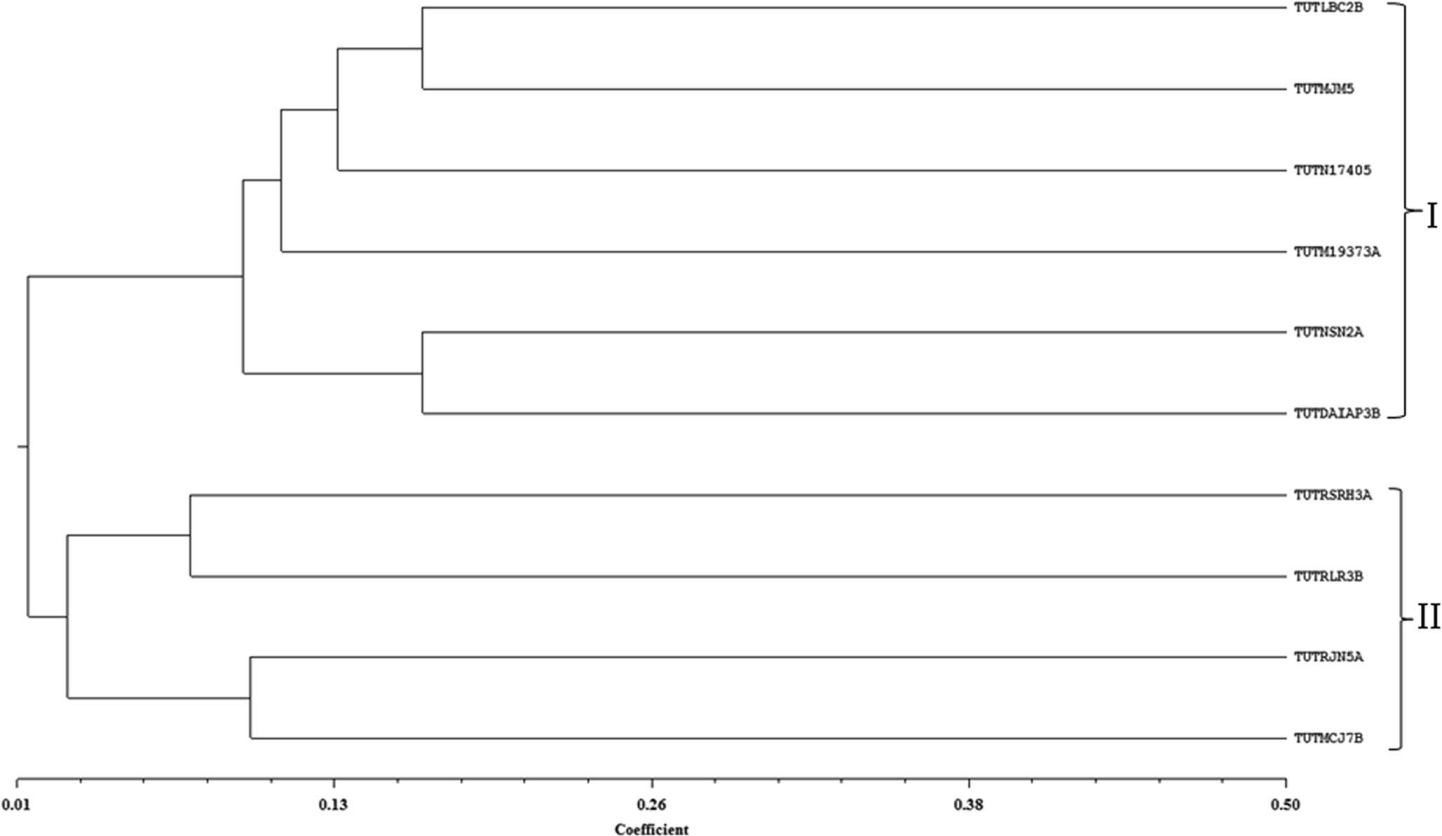
Dendrogram generated from ERIC-PCR banding pattern of soybean nodulating rhizobial isolates

The number of days to colony appearance on Yeast mannitol agar plates varied among the isolates, and ranged between 4 and 9 days (Table 1). Except for isolate TUTNSN2A which was oval, the remaining isolates were round in shape. Colony diameter also varied among isolates, ranging from <1 mm for isolates TUTRSRH3A and TUTRLR3B which were both opaque in appearance, 7 mm for isolate TUTN17405, and between 1 and 3 mm for the remaining isolates (Table 1). The genetic relationship among test rhizobial isolates based on ERIC-PCR analysis revealed the presence of highly diverse and polymorphic bands (Fig. 1). The dendrogram generated by UPGMA cluster analysis showed 2 major clusters observed at <10% Jaccard’s similarity coefficient (Fig. 2). Cluster I comprised isolates TUTLBC2B, TUTMJM5, TUTN17405, TUTM19373A, TUTNSN2A and TUTDAIAP3B while isolates TUTRSRH3A, TUTRLR3B, TUTRJN5A and TUTMCJ7B were grouped in Cluster II (Fig. 2).

### Root nodulation

All the five legume varieties tested failed to nodulate with isolate TUTLBC2B. Similarly, cowpea, Kersting’s bean and Bambara groundnut also failed to form root nodules with isolate TUTNSN2A and TUTDAIAP3B (Table 2). Although the remaining strains elicited nodulation in cowpea and Kersting’s bean, they were functionally ineffective in N_2_ fixation (Table 2). However, they formed effective nodules on Bambara groundnut and the two soybean genotypes. The two commercial *Bradyrhizobium* strains used effectively nodulated all five test genotypes. But no root nodules were found on uninoculated plants and those treated to 5 mM NO_3_^−^ (Table 2).

**Table 2:** Nodule number and nodule dry mater of five legume species inoculated with indigenous rhizobial strains in the glasshouse

Treatment	Cowpea		Bambara		Kersting’s bean		Soybean cv. Pan1664R		Soybean cv. TGx1835-10E	
	Nodule No.	Nodule DM	Nodule No.	Nodule DM	Nodule No.	Nodule DM	Nodule No.	Nodule DM	Nodule No.	Nodule DM
	per plant	mg. plant^−1^	per plant	mg. plant^−1^	per plant	mg. plant^−1^	per plant	mg. plant^−1^	per plant	mg. plant^−1^
TUTRSRH3A	157 ± 80.97ab	243.3 ± 74.5bc	127 ± 5.78abc	633.3 ± 33.3a	19 ± 1.86b	200.0 ± 0.0a	91 ± 6.35b	566.7 ± 88.19a	97 ± 11.37ab	1766.7 ± 88.19a
TUTM19373A	168 ± 43.81ab	420.0 ± 20.0a	94 ± 3.06bcd	533.3 ± 33.3a	31 ± 2.91a	200.0 ± 0.0a	114 ± 3.79a	500.0 ± 57.7ab	97 ± 7.22ab	1666.7 ± 176.4a
TUTNSN2A	0.0 ± 0.0	0.00 ± 0.0	0.0 ± 0.0	0.00 ± 00	0 ± 0.0	0.00 ± 0.0	75 ± 2.89c	300.0 ± 100.0 cd	0.0 ± 0.0	0.00 ± 0.0
TUTN17405	3 ± 0.67c	166.7 ± 66.7c	80 ± 10.17cde	333.3 ± 33.3b	0 ± 0.0	0.00 ± 0.0	58 ± 9.17d	333.3 ± 33.3 cd	78 ± 8.66bc	1766.7 ± 66.7a
TUTDAIAP3B	0.0 ± 0.0	0.0 ± 0.0	0.0 ± 0.0	0.00 ± 00	0 ± 0.0	0.00 ± 0.0	7 ± 0.58f	200.0 ± 0.00d	79 ± 4.62bc	2000 ± 57.7a
TUTRJN5A	40 ± 1.45bc	100.0 ± 0.0c	35 ± 4.33e	300.3 ± 57.7b	30 ± 5.93ab	166.7 ± 16.7b	21 ± 0.87ef	200.0 ± 0.00d	75 ± 2.89bc	1766.7 ± 88.19a
TUTMJM5	79 ± 9.39abc	130.0 ± 5.8c	66 ± 20.25de	366.7 ± 33.3b	26 ± 3.18ab	200.0 ± 0.0a	23 ± 1.20e	300.0 ± 0.00 cd	117 ± 9.33a	1500.0 ± 208.2a
TUTMCJ7B	203 ± 56.08a	333.3 ± 33.3ab	172 ± 37.69a	516.7 ± 44.1a	4 ± 0.67	200.0 ± 0.0a	58 ± 3.75d	400.0 ± 57.7bc	89 ± 0.58b	1666.7 ± 88.19a
TUTRLR3B	190 ± 34.12a	416.7 ± 93.9a	137 ± 20.42ab	533.3 ± 33.3a	26 ± 2.33ab	200.0 ± 0.0a	84 ± 0.87bc	433.3 ± 33.3abc	95 ± 0.58b	1700.0 ± 57.7a
*Bradyrhizobium*	13 ± 1.33c	433.3 ± 33.3a	26 ± 5.84e	300.0 ± 0.0b	24 ± 2.03ab	200.0 ± 0.0a	52 ± 7.45d	366.7 ± 66.7bc	58 ± 0.87c	1400.0 ± 152.8a
^a^F-statistics	4.14*	7.60***	8.71***	11.95***	7.78**	4.00*	52.61***	6.18***	6.30***	2.26 ns

^a^Treatments which did not induce nodulation were excluded from statistical analysis. Isolate TUTLBC2B failed to nodulate all test legumes and was therefore excluded from this table. The 5 mM treatment and uninoculated control were also excluded from the table

Isolate TUTMCJ7B elicited the highest number of nodules (171 per plant) on Bambara groundnut, followed by TUTRLR3B (137 nod.plant^−1^) and TUTRSRH3A (127 nod.plant^−1^). The commercial *Bradyrhizobium* strain CB765 produced the least number of nodules (26 nod.plant^−1^). With soybean, isolate TUTM19373A produced the most nodules on the genotype PAN1664R (114 nod.plant^−1^), followed by TUTRSRH3A (91 nod.plant^−1^) and TUTRLR3B (84 nod.plant^−1^). Of the effective symbioses, TUTRSRH3A produced significantly more nodule dry weight (633 mg.plant^−1^) with Bambara groundnut, followed by TUTM19373A and TUTRLR3B (533.3 mg.plant^−1^ each) and TUTMCJ7B (516.7 mg.plant^−1^). The least nodule DM was recorded by TUTRJN5A and the inoculant strain CB756 (300.0 mg.plant^−1^). With soybean, isolate TUTRSRH3A again produced the largest nodule dry mass (566.7 mg.plant^−1^) with genotype PAN1665R, followed by TUTM19373A (500 mg.plant^−1^), TUTRLR3B (433.3 mg.plant^−1^) and TUTMCJ7B (400.0 mg.plant^−1^). The lowest nodule DM was recorded by TUTDAIAP3B and TUTRJN5A (200.0 mg.plant^−1^ each) on genotype PAN1664R (Table 2). However, with soybean TGx1835-10E, TUTDAIAP3B produced much greater nodule dry matter (2000.0 mg.plant^−1^), followed by TUTRSRH3A, TUTRJN5A and TUTN17405 (1766.7 mg.plant^−1^ each). The commercial strain produced the least nodule dry matter (Table 2).

### Photosynthetic rates, stomatal conductance and leaf transpiration induced by test isolates

Cowpea nodulation by the test isolates was ineffective, except for the commercial strain CB756. As a result, photosynthetic rates and stomatal conductance were markedly higher for strain CB756 relative to the other isolates (Table 3). The 5 mM NO_3_-fed cowpea seedlings also recorded high photosynthetic rates and stomatal conductance. Water loss by leaf transpiration was higher in plants nodulated by *Bradyrhizobium* strain CB756 as well as in NO_3_-fed cowpea plants (Table 3).

**Table 3:** Photosynthetic rates (A) rates, stomatal conductance (gs) and leaf transpiration (E) induced by soybean rhizobial isolates on four grain legumes

Treatment	Cowpea			Bambara			Kersting’s bean			Soybean cv. PAN1664R			Soybean cv. TGx1835-10E		
	A	gs	E	A	gs	E	A	gs	E	A	gs	E	A	gs	E
	μmol (CO_2_) m^−2^ s^−1^	mol (H_2_O) m^−2^ s^−1^	mol (H_2_O) ms^−2^ s^−1^	μmol (CO_2_) m^−2^ s^−1^	mol (H_2_O) m^−2^ s^−1^	mol (H_2_O) ms^−2^ s^−1^	μmol (CO_2_) m^−2^ s^−1^	mol (H_2_O) m^−2^ s^−1^	mol (H_2_O) ms^−2^ s^−1^	μmol (CO_2_) m^−2^ s^−1^	mol (H_2_O) m^−2^ s^−1^	mol (H_2_O) ms^−2^ s^−1^	μmol (CO_2_) m^−2^ s^−1^	mol (H_2_O) m^−2^ s^−1^	mol (H_2_O) ms^−2^ s^−1^
TUTLBC2B	1.91 ± 0.53 fg	0.01 ± 0.00 fg	0.55 ± 0.11 g	5.6 ± 0.36e	0.032 ± 0.00f	0.81 ± 0.07f	1.09 ± 0.03f	0.017 ± 0.00c	0.76 ± 0.00d	3.8 ± 0.02de	0.03 ± 0.00 g	1.12 ± 0.00f	3.3 ± 0.04f	0.025 ± 0.00d	0.92 ± 0.00e
TUTRSRH3A	2.04 ± 0.03 fg	0.01 ± 0.00 fg	0.41 ± 0.00 h	17.0 ± 2.80a	0.135 ± 0.03b	3.0 ± 0.60c	3.20 ± 0.01c	0.024 ± 0.00b	1.07 ± 0.01b	15.6 ± 0.01a	0.16 ± 0.00b	5.50 ± 0.00a	14.1 ± 0.03c	0.094 ± 0.00bc	3.03 ± 0.01 cd
TUTM19373A	7.43 ± 1.13d	0.04 ± 0.01de	1.44 ± 0.10d	15.0 ± 0.02ab	0.10 ± 0.003c	2.5 ± 0.03d	4.24 ± 0.03a	0.029 ± 0.00a	1.24 ± 0.00a	16.5 ± 1.5a	0.13 ± 0.02c	4.50 ± 0.65b	14.4 ± 0.03c	0.12 ± 0.00ab	3.14 ± 0.02bcd
TUTNSN2A	2.20 ± 0.03f	0.02 ± 0.00f	0.56 ± 0.00 g	5.4 ± 0.02e	0.03 ± 0.00f	0.83 ± 0.00	0.68 ± 0.02 g	0.009 ± 0.00f	0.39 ± 0.01 h	9.7 ± 0.06c	0.05 ± 0.00ef	1.86 ± 0.01e	3.1 ± 0.07f	0.03 ± 0.00d	1.17 ± 0.08e
TUTN17405	2.46 ± 0.01f	0.02 ± 0.00 fg	0.62 ± 0.00 g	11.2 ± 0.04 cd	0.08 ± 0.00de	1.90 ± 0.01f	0.75 ± 0.01 g	0.013 ± 0.00d	0.52 ± 0.00f	13.0 ± 0.03b	0.19 ± 0.00a	5.75 ± 0.01a	16.2 ± 1.5ab	0.13 ± 0.03a	4.01 ± 0.65a
TUTDAIAP3B	2.26 ± 0.02f	0.02 ± 0.00f	0.53 ± 0.00 gh	4.6 ± 0.02e	0.02 ± 0.001f	0.56 ± 0.00f	2.24 ± 0.11d	0.023 ± 0.00b	0.94 ± 0.02c	3.0 ± 0.03e	0.03 ± 0.00 fg	1.27 ± 0.00ef	11.6 ± 0.08d	0.08 ± 0.00c	2.45 ± 0.01d
TUTRJN5A	1.04 ± 0.04 g	0.01 ± 0.00 g	0.24 ± 0.00i	9.1 ± 0.04d	0.06 ± 0.001e	1.75 ± 0.01e	2.05 ± 0.04d	0.013 ± 0.00d	0.59 ± 0.00e	5.2 ± 0.05d	0.07 ± 0.00e	2.76 ± 0.06d	15.0 ± 0.04bc	0.11 ± 0.00abc	3.16 ± 0.01bcd
TUTMJM5	2.40 ± 0.01f	0.02 ± 0.00f	0.51 ± 0.00 gh	14.1 ± 0.03b	0.12 ± 0.00bc	2.86 ± 0.02 cd	2.08 ± 0.25d	0.013 ± 0.00d	0.56 ± 0.05ef	16.5 ± 0.01a	0.19 ± 0.00a	5.74 ± 0.02a	11.3 ± 0.9d	0.08 ± 0.01c	2.60 ± 0.45d
TUTMCJ7B	7.00 ± 0.06d	0.05 ± 0.00d	1.25 ± 0.01e	11.6 ± 0.08c	0.10 ± 0.00 cd	2.47 ± 0.01d	1.42 ± 0.04e	0.011 ± 0.00e	0.46 ± 0.00 g	16.5 ± 0.04a	0.19 ± 0.00a	5.80 ± 0.03a	17.2 ± 1.1a	0.14 ± 0.02a	4.37 ± 0.45a
TUTRLR3B	13.85 ± 0.08b	0.10 ± 0.00b	2.54 ± 0.01b	15.3 ± 0.01ab	0.11 ± 0.00bc	2.64 ± 0.02 cd	1.01 ± 0.04f	0.006 ± 0.00 g	0.23 ± 0.00i	16.5 ± 0.02a	0.16 ± 0.00b	5.32 ± 0.01a	13.9 ± 0.02c	0.11 ± 0.00ab	3.69 ± 0.01abc
*Bradyrhizobium*	23.29 ± 0.01a	0.22 ± 0.00a	4.47 ± 0.06a	17.1 ± 0.04a	0.21 ± 0.00a	5.52 ± 0.01a	2.30 ± 0.04d	0.024 ± 0.00b	0.91 ± 0.00c	15.7 ± 1.3a	0.16 ± 0.02b	5.09 ± 0.46ab	14.5 ± 0.05bc	0.11 ± 0.00abc	3.64 ± 0.00abc
5 mM KNO_3_	9.46 ± 0.04c	0.06 ± 0.00c	1.66 ± 0.01c	15.0 ± 0.06ab	0.19 ± 0.00a	3.64 ± 0.02b	3.91 ± 0.08b	0.029 ± 0.00a	1.21 ± 0.01a	9.6 ± 0.02c	0.10 ± 0.00d	3.51 ± 0.01c	8.9 ± 0.01e	0.11 ± 0.00ab	3.91 ± 0.00ab
Uninoc control	4.74 ± 0.17e	0.04 ± 0.00e	1.09 ± 0.00f	4.5 ± 0.04e	0.02 ± 0.00f	0.62 ± 0.00f	1.12 ± 0.04f	0.005 ± 0.00 g	0.24 ± 0.00i	4.0 ± 0.44de	0.03 ± 0.00 g	1.11 ± 0.16f	4.6 ± 0.02f	0.04 ± 0.00d	1.66 ± 0.00e
F-statistics	330.2**	553.7**	**	38.18***	52.78***	134.14**	195.5**	**	434.5**	93.51**	72.34***	71.51***	72.45***	14.86***	10.26***

In general, the plants that were not nodulated or ineffectively nodulated revealed much lower photosynthesis rates and stomatal conductance. Cowpea and Kersting’s groundnut plants all showed very low photosynthetic and stomatal activity due to their poor nodulation status (Table 3).

Of the effectively nodulated Bambara groundnut plants, isolate TUTRSRH3A and strain CB756 elicited greater photosynthetic rates in leaves, followed by TUTRLR3B, TUTM19373A and TUTMJM5, and the 5 mM NO_3_-fed plants (Table 3). As a result, stomatal conductance was also increased by these strains and 5 mM NO_3_-feeding.

With soybean, isolates TUTM19373A, TUTMJM5, TUTMCJ7B and TUTRLR3B again induced greater photosynthetic activity in leaves of genotype PAN1664R, followed by strain CB756 and TUTRSRH3A (Table 3). As a result, stomatal functioning was highly increased in plants nodulated by isolates TUTMJM5 and TUTMCJ7B followed by TUTRSRH3A, TUTRLR3B and strain WB74 and TUTM19373A (Table 3).

However, isolates TUTRSRH3A, TUTN17405, TUTMJM5, TUTMCJ7B and TUTRLR3B elicited much greater water loss via leaf transpiration in PAN166R followed by strain WB74 (Table 3). With soybean genotype TGx1835-10E, the highest photosynthetic rates and stomatal functioning were induced by isolates TUTMCJ7B, TUT17405 and TUTRJN5A (Table 3), which led to increased water loss through leaf transpiration (Table 3).

### Shoot and whole-plant biomass

Of the effective Bambara strain symbioses, isolate TUTRSRH3A caused greater accumulation of shoot biomass (2.9 g.plant^−1^), followed by the 5 mM NO_3_-fed plants. With soybean, TUTM19373A produced much greater shoot DM (4.0 g.plant^−1^) with PAN1664R, followed by TUTRSRH3A (3.7 g.plant^−1^) and TUTRLR3B (3.3 g.plant^−1^). However, TUTRLR3B accumulated the most shoot biomass with TGx1835-10E, followed by TUTRJN5A and TUTMCJ7B (3.1 g.plant^−1^ each). The commercial strain WB74 and 5 mM NO_3_-fed plants of genotype TGx1835-10E recorded less shoot biomass than the top performing three isolates (Table 4).

**Table 4:** Shoot dry matter (shoot DM) and N concentration of five legumes species inoculated with indigenous rhizobial strains in the glasshouse

Treatment	Cowpea		Bambara		Kersting’s bean		Soybean cv. Pan1664R		Soybean cv. TGx1835-10E	
	Shoot DM	Shoot N conc’n	Shoot DM	Shoot N conc’n	Shoot DM	Shoot N conc’n	Shoot DM	Shoot N conc’n	Shoot DM	Shoot N conc’n
	g. plant^−1^	%	g. plant^−1^	%	g. plant^−1^	%	g. plant^−1^	%	g. plant^−1^	%
TUTLBC2B	0.4 ± 0.0def	0.8 ± 0.0 cdef	1.5 ± 0.2de	0.97 ± 0.06bcb	0.3 ± 0.0de	0.8 ± 0.1ab	1.1 ± 0.1de	0.9 ± 0.0ef	0.9 ± 0.1ef	0.80 ± 0.07ef
TUTRSRH3A	0.5 ± 0.1 cde	0.9 ± 0.1 cde	2.9 ± 0.3a	1.32 ± 0.06bcd	0.2 ± 0.1de	0.9 ± 0.0ab	3.7 ± 0.1ab	2.3 ± 0.3 cd	2.8 ± 0.3ab	2.23 ± 0.17bc
TUTM19373A	1.0 ± 0.1b	0.9 ± 0.0c	2.1 ± 0.1bc	1.40 ± 0.08abc	0.3 ± 0.0 cd	0.9 ± 0.0b	4.0 ± 0.2a	2.6 ± 0.2bcd	3.0 ± 0.3ab	2.02 ± 0.34c
TUTNSN2A	0.3 ± 0.0ef	0.7 ± 0.0f	1.4 ± 0.1de	0.99 ± 0.08bcd	0.3 ± 0.0d	0.7 ± 0.0c	1.8 ± 0.0ef	1.4 ± 0.3e	1.0 ± 0.1f	0.59 ± 0.04f
TUTN17405	0.5 ± 0.0 cde	0.7 ± 0.0f	1.7 ± 0.1 cd	1.03 ± 0.04bcd	0.3 ± 0.0 cd	0.8 ± 0.1ab	2.5 ± 0.1c	2.3 ± 0.1 cd	2.5 ± 0.2bc	2.45 ± 0.04bc
TUTDAIAP3B	0.4 ± 0.0def	0.7 ± 0.0def	1.4 ± 0.0de	0.89 ± 0.04d	0.2 ± 0.0e	0.9 ± 0.1ab	0.4 ± 0.1f	0.8 ± 0.2f	2.7 ± 0.4abc	2.11 ± 0.03bc
TUTRJN5A	0.2 ± 0.0f	0.7 ± 0.1ef	1.9 ± 0.3e	1.13 ± 0.05 cd	0.4 ± 0.0b	0.9 ± 0.0ab	0.9 ± 0.0ef	2.3 ± 0.2d	3.1 ± 0.3ab	2.39 ± 0.08bc
TUTMJM5	0.2 ± 0.1f	0.9 ± 0.0 cd	2.2 ± 0.2de	1.27 ± 0.02bcd	0.4 ± 0.1bc	0.9 ± 0.0b	2.0 ± 0.1d	2.4 ± 0.3bcd	2.7 ± 0.5abc	2.45 ± 0.16bc
TUTMCJ7B	1.1 ± 0.0b	0.9 ± 0.0c	2.1 ± 0.4bc	1.44 ± 0.13ab	0.3 ± 0.0d	0.8 ± 0.0ab	2.9 ± 0.2c	3.0 ± 0.1b	3.1 ± 0.1ab	2.55 ± 0.09ab
TUTRLR3B	0.6 ± 0.2 cd	0.7 ± 0.0ef	2.2 ± 0.1bc	1.40 ± 0.01abc	0.3 ± 0.0de	0.9 ± 0.1ab	3.3 ± 0.1b	2.9 ± 0.3bc	3.4 ± 0.5a	2.31 ± 0.26bc
*Bradyrhizobium*	1.5 ± 0.2a	1.7 ± 0.1a	2.8 ± 0.1de	1.81 ± 0.45a	0.5 ± 0.1b	0.9 ± 0.0b	2.5 ± 0.31c	3.8 ± 0.2a	2.5 ± 0.2bc	3.03 ± 0.16a
5 mM KNO_3_	1.2 ± 0.1b	1.4 ± 0.1b	2.6 ± 0.2ab	1.39 ± 0.14abc	1.0 ± 0.0a	1.2 ± 0.0a	2.7 ± 0.2c	1.0 ± 0.1ef	1.9 ± 0.0 cd	1.37 ± 0.30d
Uninoc control	0.7 ± 0.0c	1.0 ± 0.1c	1.1 ± 0.1e	0.97 ± 0.03bcd	0.3 ± 0.0 cd	0.8 ± 0.1ab	1.0 ± 0.1e	0.8 ± 0.0ef	1.2 ± 0.1de	1.08 ± 0.01de
F-statistics	28.4***	29.4***	10.71***	3.8**	33.7***	4.2**	73.7***	22.9***	14.2***	19.3***

Dry matter accumulation at whole-plant level also varied with isolates. As shown in Table 5, isolate TUTRSRH3A induced greater accumulation of dry matter in whole plants of Bambara groundnut, followed by strain CB756, TUTMCJ7B and TUTRLR3B. Isolates TUTM19373A and TUTRSRH3A produced the most biomass in whole plants of soybean genotype PAN1664R, followed by TUTMCJ7B, TUTRLR3B and the commercial strain WB74, while with the TGx1836-10E soybean, isolate TUTRLR3B induced greater accumulation of whole-plant biomass, followed by TUTRSRH3A, TUTM19373A, TUTRJN5A, TUTMCJ7B and TUTDAIAP3B (Table 5).

**Table 5:** Whole-plant (WP) dry matter (shoot + root + nodule) and N-fixed in three legumes species effectively nodulated by indigenous soybean rhizobial strains

Treatment	Bambara		Soybean cv. PAN1664R		Soybean cv. TGx1835-10E	
	WP DM	WP N-fixed	WP DM	WP N-fixed	WP DM	WP N-fixed
	g. plant^−1^	mg. plant^−1^	g. plant^−1^	mg. plant^−1^	g. plant^−1^	mg. plant^−1^
TUTLBC2B	2.13 ± 0.20def	–	1.73 ± 0.09d	–	0.73 ± 0.07d	–
TUTRSRH3A	4.23 ± 0.26a	55.72 ± 3.93a	5.03 ± 0.22a	115.98 ± 10.80b	5.80 ± 0.38ab	128.94 ± 9.56bc
TUTM19373A	3.30 ± 0.10bc	46.33 ± 3.80ab	5.30 ± 0.17a	135.86 ± 6.35ab	5.73 ± 0.58ab	118.31 ± 27.93 cd
TUTNSN2A	1.90 ± 0.17ef	–	1.60 ± 0.12d	23.20 ± 5.76d	0.60 ± 0.12d	–
TUTN17405	2.70 ± 0.21 cd	27.87 ± 3.15bc	2.10 ± 0.06d	48.89 ± 2.88c	4.83 ± 0.28b	118.73 ± 8.38 cd
TUTDAIAP3B	1.90 ± 0.00ef	–	2.60 ± 1.45 cd	16.71 ± 6.50d	5.10 ± 0.71ab	107.83 ± 15.15d
TUTRJN5A	2.43 ± 0.03de	22.73 ± 1.31c	1.57 ± 0.03d	35.26 ± 3.26 cd	5.83 ± 0.35ab	138.69 ± 3.93ab
TUTMJM5	2.17 ± 0.03def	27.52 ± 0.57bc	2.27 ± 0.09d	53.49 ± 5.93c	4.97 ± 0.65b	120.49 ± 11.42 cd
TUTMCJ7B	3.52 ± 0.51b	50.57 ± 7.65a	4.17 ± 0.12ab	124.18 ± 4.02b	5.70 ± 0.10ab	145.24 ± 4.09a
TUTRLR3B	3.43 ± 0.20b	48.08 ± 2.97a	4.73 ± 0.12ab	137.88 ± 9.68ab	6.47 ± 0.92a	146.81 ± 17.07a
*Bradyrhizobium*	3.67 ± 0.12ab	65.30 ± 14.54a	4.17 ± 0.33ab	155.83 ± 12.54a	4.97 ± 0.38b	149.40 ± 7.40a
5 mM KNO_3_	3.23 ± 0.26bc	na	3.67 ± 0.30bc	na	2.73 ± 0.12c	na
Uninoc control	1.60 ± 0.06f	–	1.37 ± 0.12d	–	1.70 ± 0.15 cd	–
F statistics	15.38***	5.82**	11.12***	52.49***	20.58***	8.47***

The legume species showing ineffective nodulation were excluded in this analysis. na = not applicable, − = no nodules

### Shoot N concentration

*Bradyrhizobium* strain CB756 effectively nodulated cowpea and therefore significantly increased its shoot N concentration compared to plants ineffectively nodulated by the test isolates (Table 4). The 5 mM NO_3_-fed plants showed the next highest shoot N concentration. Of the effectively nodulated Bambara groundnut, strain CB756 produced significantly more shoot N concentration, followed by TUTMCJ7B, then TUTRLR3B and TUTM19373A (Table 4). With soybean genotypes PAN1664R and TGx183510-E, the commercial strain caused greater N concentration in shoots, followed by TUTMCJ7B and TUTRLR3B (Table 4). Shoot N levels were much lower in the 5 mM NO_3_-fed plants compared to the best performing strains.

### Amount of N-fixed

The amount of N-fixed per whole plant of Bambara groundnut was highest with strain CB 756, TUTRSRH3A, TUTMCJ7B and TUTRLR3B, but lowest with TUTRJN5A (Table 5). Similarly, at whole-plant level, strain WB74, TUTRLR3B, and TUT19373A produced similar but much higher amounts of symbiotic N in soybean genotype PAN1664R, followed by TUTMCJ7B and TUTRSRH3A (Table 5). Isolate TUTDAIAP3B fixed the lowest amount of N with genotype PAN1664R. Commercial strain WB74 and isolates TUTRLR3B and TUTMCJ7B fixed similar but markedly increased amounts of N in genotype TGx1835-10E. Isolate TUTRJN5A, which fixed the least N with PAN1664R, fixed similar levels as the top performing strains (Table 5).

## Discussion

Cross-infectivity assay of native isolates on different legume species is the first step to determining host range, and hence strain potential for use as inoculant. In this study, ten soybean rhizobia native to South African and Mozambican soils were evaluated for their ability to elicit nodulation in cowpea, Bambara groundnuts, Kersting’s groundnut and soybean (the homologous host). All test isolates were highly diverse based on colony morphology and ERIC-PCR fingerprinting. One isolate (TUTLBC2B) failed to cause nodulation in all test species, and two isolates (TUTLBC2B and TUTDAIAP3B) did not induce nodule formation in cowpea, Bambara groundnut and Kersting’s bean (Table 2). The remaining seven isolates all caused ineffective nodulation in cowpea, but induced effective N_2_-fixing nodules in Bambara groundnut (Table 2). These results contrast those of Musiyiwa et al. (2005) who found soybean rhizobial isolates to nodulate effectively with cowpea, and ineffectively with Bambara groundnut. But what was even more intriguing is the fact that TUTMJM5, TUTMCJ7B and TUTRLR3B, which are *B. elkanii* strains, failed to effectively nodulate cowpea even though *Bradyrhizobium elkanii* is the dominant microsymbiont nodulating soybean in Mozambique, and has been isolated from cowpea root nodules in that country (data not shown). These findings suggest that microsymbiont relatedness with standard reference strains in phylogenetic analysis should be confirmed through nodulation studies under glasshouse conditions. However, the fact that seven of the ten test isolates effectively nodulated Bambara groundnut was not surprising as Doku (1969) earlier showed that nodule bacteria from cowpea, soybean, lima bean and groundnut could effectively nodulate and fix N_2_ in Bambara groundnut.

The N_2_-fixing isolates did not only produce varied nodule numbers and nodule dry weights with their host plants (Table 2), but also different levels of N_2_ fixation (measured here as shoot N concentration and amounts of N-fixed). In general, the isolates that formed the most root nodules per plant also produced greater nodule dry weights (Tables 2). In this study, nodulation appeared to closely mirror whole-plant biomass and amount of N-fixed. As shown in Table 2, bacterial stimulation of nodule numbers and nodule dry weights was generally greater with isolates TUTRSRH3A, TUTM19373A, TUTMCJ7B, TUTRLR3B and TUTRJN5A. As a result, shoot and whole-pant dry matter were also generally increased in Bambara groundnut and the two soybean genotypes that formed effective symbioses with the isolates (Tables 4 and 5).

Gas-exchange studies revealed relatively lower photosynthetic rates, stomatal conductance and leaf transpiration in the non-nodulated and ineffectively nodulated plants of all the test legume genotypes (Table 3). In contrast, effectively nodulated plants of Bambara groundnut and the two soybean genotypes showed significantly increased photosynthesis and stomatal functioning (Table 3). But more specifically, effective nodulation of Bambara groundnut by strain CB756, TUTRSRH3A, TUTRLR3B and TUTM19373A markedly raised photosynthetic rates and stomatal functioning than the other isolates. Similarly, leaf photosynthetic activity, stomatal conductance and transpiration were much greater in the two soybean genotypes when nodulated by TUTMCJ7B, TUTRLR3B, commercial strain WB74 and TUTMJM5 (Table 3). As to be expected, the increased photosynthesis induced by these better performing rhizobial isolates led to greater accumulation of shoot and whole-plant dry matter (Tables 4 and 5).

**Fig. 3:**
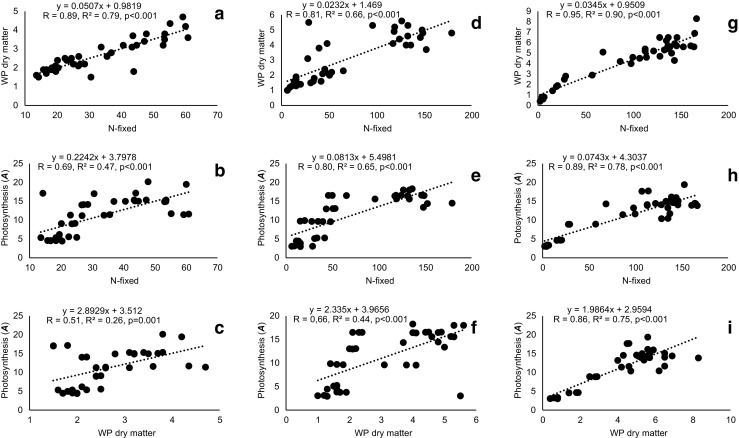
Correlations between (**a**) N-fixed and whole plant (WP) dry matter of Bambara groundnut, (**b**) N-fixed and ***A*** of Bambara groundnut, (**c**) WP dry matter and ***A*** of Bambara groundnut, (**d**) N-fixed and whole plant (WP) dry matter of Soybean (PAN1664R), (**e**) N-fixed and ***A*** of PAN1664R, (**f**) WP dry matter and ***A*** of PAN1664R as well as between (**g**) N-fixed and WP dry matter of Soybean (TGx1835-10E, **h**) N-fixed and ***A*** of TGx1835-10E and **i**) WP dry matter and ***A*** of TGx1835-10E planted in the glasshouse

However, because legume N_2_ fixation is driven by de novo products of photosynthesis, the rhizobial isolates that elicited higher photosynthetic activity also stimulated greater N_2_ fixation, and increased the amounts of N-fixed at whole-plant level (Table 5). In essence, the C sink strength of the different host/strain symbioses differed according to the isolate’s compatibility and N_2_-fixing efficiency with its specific host legume. That de novo photosynthate from legume leaves enhanced N_2_ fixation, which in turn provided more symbiotic N for Rubisco biosynthesis, and hence increased photosynthesis and C accumulation as biomass, is supported by the correlation/regression analyses presented in Fig. 3. In fact, for all the effectively functional Bambara groundnut symbioses with test rhizobial isolates, there was a highly significant correlation between photosynthesis and N-fixed (Fig. 3a), photosynthesis and dry matter yield (Fig. 3b), as well as N-fixed and whole-plant biomass (Fig. 3c). Similar relationships were found for the soybean symbioses involving genotypes PAN1664R (Fig. 3d-f) and TGx1835-10E (Fig. 3g-i). Therefore, the C sink strength of rhizobial symbioses is the main driver of plant growth, dry matter accumulation and symbiotic performance in nodulated legumes (Kaschuk et al. 2009).

A closer scrutiny of treatment effects showed that TUTRSRH3A, strain CB756 and NO_3_-feeding caused significantly greater accumulation of biomass in Bambara groundnut (Tables 4 and 5). Similarly, isolates TUTM19373A, TUTRSRH3A and TUTRLR3B were found to induce markedly increased shoot and whole-plant dry matter in soybean genotype PAN1664R, in a manner similar to strains TUTRLR3B and TUTM19373A which also elicited greater shoot and whole-plant biomass in genotype TGx1835-10E (Tables 4 and 5). In terms of symbiotic performance, strain CB756 caused the highest shoot N concentration and amount of N-fixed in Bambara groundnut, followed by TUTMCJ7B, and then TUTM19373A and TUTRLR3B which effected similar shoot N levels (Tables 4 and 5). With soybean genotype PAN1664R, nodulation by *B. japonicum* strain WB74, and isolates TUTRLR3B, TUTM119373A, TUTMCJ7B and TUTRSRH3A (in that order) markedly increased shoot N concentrations and amounts of N-fixed when compared to the remaining test strains (Tables 4 and 5). However, nodulation of TGx1835-10E by *B. japonicum* WB74, TUTRLR3B, TUTMCJ7B and TUTRJN5A also significantly increased fixed-N levels relative to the other isolates (Table 5).

Whether based on Bambara groundnut nodulation, or that of the two soybean genotypes, whole-plant symbiotic N was highest for the commercial strains CB756 (Bambara groundnut) and WB74 (soybean), as well as for TUTRLR3B, TUTMCJ7B and TUTRSRH3A when compared to the other isolates (Table 5). This suggests that the native rhizobial isolates TUTRLR3B, TUTMCJ7B and TUTRSRH3A have potential for use as inoculants.
